# Polarity-Aware Knowledge Graph Reveals Diet-Microbiome-Health Mechanisms with Relevance to Muscle, Immune and Metabolic Aging

**DOI:** 10.21203/rs.3.rs-8771108/v1

**Published:** 2026-02-16

**Authors:** Jianfu Li, Ellen Bu, Andrew Lian, Yiming Li, Cui Tao

**Affiliations:** 1 Department of Artificial Intelligence and Informatics, Mayo Clinic, Jacksonville, FL 32224, USA; 2 Richard Montgomery High School, Rockville, MD 20852, USA; 3 Department of Psychology, University of Southern California, Los Angeles, CA 90089, USA; 4 McWilliams School of Biomedical Informatics, University of Texas Health Science Center at Houston, Houston, TX 77030, USA

## Abstract

Diet profoundly influences gut microbial composition and metabolism, yet mechanistic pathways linking dietary exposures to human health remain fragmented across the literature. To systematically organize and integrate this evidence, we constructed an evidence-weighted Diet-Microbiome-Health Knowledge Graph (DMH-KG) from 1,309 curated PubMed abstracts (2023–2025), using a standardized schema spanning 11 entity categories and 12 relationship types with explicit polarity labels. Entities and relationships were identified through manual annotation, yielding 10,270 entity mentions and 4,866 relationships. Expert-guided consolidation of synonymous and lexically variant terms, along with pruning of disconnected concepts, resulted in 4,766 unique entities connected by 4,772 polarity-weighted edges. To prioritize robust biological signals, a composite edge-weighting function was applied, integrating both relationship frequency and polarity. Network analysis revealed a modular small-world structure centered on microbial and inflammatory mediators. High-confidence pathways emerged, including the probiotic-SCFA-immunity axis (34 supporting documents) and the high-fat diet-LPS-endotoxemia cascade (8 documents), both of which are central to age-related immune modulation and metabolic health. Quantitative validation against five KEGG and Reactome pathways demonstrated high biological fidelity: the DMH-KG recovered all reference diet-microbiome-outcome edges for Butanoate metabolism and Secondary Bile Acid biosynthesis (100% coverage) and achieved a mean pathway-level entity coverage of 92.0%, measured as the proportion of predefined pathway components represented in the graph. A comparative pilot study further demonstrated that DMH-KG augmentation improves mechanistic specificity inference across Diet-Microbiome-Health interactions. These features position the DMH-KG as a scalable platform for mechanistic inference across Diet-Microbiome-Health interactions, with direct relevance to immune regulation, muscle health, metabolic aging, and chronic disease prevention. The framework preserves evidence provenance, relationship polarity, and biological direction, supporting both discovery science and AI-driven nutritional reasoning.

## Introduction

The human gut microbiome is increasingly recognized as a functional organ-like system that orchestrates host physiology, regulating essential processes ranging from metabolic homeostasis and immune ontogeny to neuroendocrine signaling^1,2^. Dysbiosis has now been implicated in the etiology of chronic conditions ranging from inflammatory bowel disease (IBD)^3^ to neurodegenerative disorders like Alzheimer’s disease via the gut-brain axis^4^. Among health-related behaviors, diet functions as the most potent and rapidly acting modulator of the microbiome. Controlled feeding studies demonstrate that dietary shifts can reshape microbial community structure and metabolic output within 24 to 48 hours, highlighting the dynamic and responsive nature of the diet-microbiome-host axis^5^. Yet large cohorts such as PREDICT-1 demonstrate that inter-individual variability in microbial composition often exceeds genetic influences in determining metabolic responses to food^6,7^. Compounding this complexity, persistent discrepancies between recommended and actual nutrient intakes, such as the well-characterized “fiber gap”, continue to drive dysbiosis and metabolic dysfunction at the population level^8^.

Despite the fundamental role of diet-microbiome interactions, mechanistic pathways remain fragmented, inconsistently reported, or only partially described across thousands of published studies. Many findings are correlative or context-dependent, and systematic reviews struggle to keep pace with the rapidly expanding literature^5,9,10^. Traditional computational methods provide taxonomic details but rarely connect these shifts to downstream biological effects. Conversely, deep learning models and LLMs often lack explicit causal grounding, leading to plausible yet mechanistically unsupported explanations^11–13^.

Knowledge graphs (KGs) offer a principled solution by transforming unstructured literature into structured, semantically coherent, and mechanistic representations of biological knowledge. Prior biomedical KGs have demonstrated value in proteomics interpretation, disease association mapping, and multimodal data integration^14,15^. When aligned with standard ontologies, they enable reasoning over multi-step causal chains rather than isolated associations, and support semantic interoperability across heterogeneous datasets^16^. This capability is particularly important in diet-microbiome research, where dietary exposures propagate through layered biological cascades—from nutrient intake to microbial metabolism to host signaling—and intersect with pathways central to immune function and metabolic aging. KGs explicitly encode these sequential dependencies, allowing mechanistic inference that traditional pairwise models cannot provide. Recent efforts, such as the construction of AcuKG^17^ for medical acupuncture, illustrate how curated knowledge graphs can integrate heterogeneous evidence into unified, interpretable frameworks that support mechanistic discovery and clinical reasoning.

Nevertheless, existing attempts to computationally synthesize this domain remain limited. Many rely on fully automated extraction pipelines, including recent neural models, which offer scalability but can introduce noise or imprecise mechanistic assertions. Other efforts lack polarity distinctions (beneficial, harmful, or neutral), which are essential for actionable nutritional inference^1,2,18^. As LLMs increasingly enter biomedical and clinical workflows, structured, provenance-linked resources are required to guide generative outputs and ensure factual reliability^12,13^.

In this study, we developed an evidence-weighted Diet-Microbiome-Health Knowledge Graph (DMH-KG) constructed from 1,309 selected PubMed abstracts, annotated using a domain-specific schema encompassing 11 entity categories and 12 directional relation types. The resulting graph integrates nutritional components, microbial taxa, metabolites, physiological processes, and disease endpoints into a unified mechanistic network. A key methodological contribution is a transparent polarity- and frequency-based edge weighting strategy that distinguishes well-supported biological signals from sparse or ambiguous findings.

Our goals were threefold:

1. To establish a transparent, domain-precise framework for synthesizing mechanistic evidence from heterogeneous diet-microbiome literature.
2. To characterize the structural and functional topology of the DMH-KG, including hubs, communities, and high-confidence mechanistic pathways.
3. To evaluate biological fidelity through quantitative reconstruction of curated KEGG and Reactome pathways, assessing how well normalized mechanisms emerge from unstructured text.

These advances provide a robust and interpretable foundation for mechanistic reasoning in nutrition science. By preserving relationship polarity, evidence provenance, and biological direction, the framework supports neurosymbolic AI systems that guide generative models in verifiable biological evidence.

## Results

### Entity Distribution and Semantic Landscape

The annotation process captured 10,270 entity mentions across the 1309-abstract corpus. The resulting distribution reflected dominant themes in diet-microbiome-health research: nutrition-related entities constituted the largest proportion (29.9%; n=3,071), followed by microbiome taxa and metabolites (23.9%; n=2,452), clinical outcomes (17.0%; n=1,745), and disease entities (15.6%; n=1,598). Expert-guided consolidation unified 599 mentions (5.8%) into 32 normalized forms, reducing lexical fragmentation and improving coherence across conceptually equivalent terms. After removal of entities not participating in any relationships, the final DMH-KG comprised 4,766 unique entities connected by 4,772 weighted edges.

**Figure 1.**
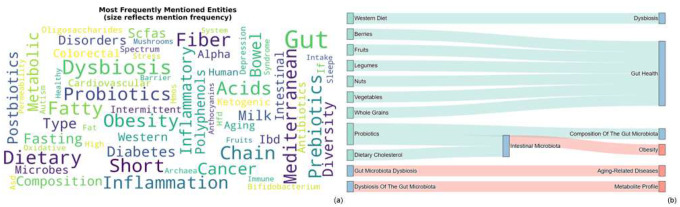
Entity distribution and semantic landscape of the DMH-KG. (a) *Entity frequency word cloud*. Entity size corresponds to relationship participation frequency. Highly represented concepts—such as probiotics (165 relationships), dysbiosis (98), and obesity (84)—serve as central hubs within the diet–microbiome–health axis in recent literature (2023–2025). (b) Sankey visualization illustrating how dietary exposures (left, cyan) co-occur with microbial mediators (middle) and downstream health outcomes (right, coral). Flow width reflects composite evidence weighting. Beneficial dietary patterns (e.g., berries, fruits, legumes, nuts, vegetables, whole grains) cluster around gut-supportive pathways, whereas dysbiosis-associated patterns align with adverse outcomes such as obesity and aging-related conditions.

Nutritional entities such as dietary fiber, polyphenols, probiotics, and short-chain fatty acids (SCFAs) appeared most frequently in the corpus. Prominent microbial taxa included Faecalibacterium prausnitzii, Akkermansia muciniphila, Lactobacillus, and Bifidobacterium, consistent with their established roles in metabolic and immune regulation. These high-frequency nodes form the primary semantic anchors of the DMH-KG. Figure 1 illustrates this landscape: the word cloud (Figure 1a) highlights recurrent metabolic and inflammatory concepts, while co-occurrence flows (Figure 1b) reveal characteristic mechanistic constellations linking dietary inputs to microbial mediators and host outcomes. Patterns such as fiber fermentation involving Faecalibacterium and Bifidobacterium, red-meat-associated carnitine metabolism by Prevotella leading to TMAO production, and probiotic-associated immunomodulation exemplify the multi-step interactions consistently reported in the literature.

### Relationship Patterns and Edge Weighting

**Figure 2.**
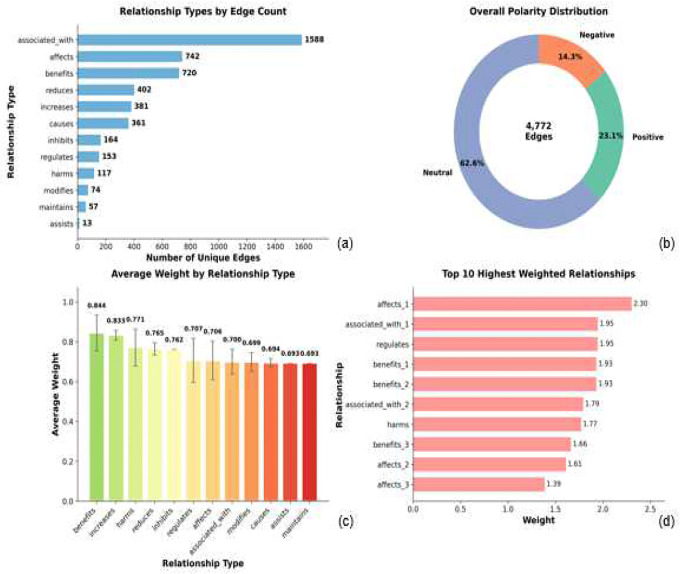
Comprehensive relationship analysis of the DMH-KG. Twelve distinct relationship types characterize the 4,772 weighted edges in the knowledge graph. (a) All relationship types ranked by edge count, showing the predominance of associated_with (n = 1,588) and the distribution across three polarity categories: neutral (62.6%), beneficial (23.1%), and harmful (14.3%). (b) Overall polarity distribution reveals that neutral/observational relationships dominate, followed by beneficial and harmful effects. (c) Average weight by relationship type demonstrates that beneficial and harmful relationships (benefits = 0.844, increases = 0.833, harms = 0.771) exhibit higher evidence strength than neutral associations (associated_with = 0.700, modifies = 0.699). (d) Top 10 highestweighted individual relationships, with suffixes (_1, _2, _3) distinguishing multiple instances of the same relationship type. Edge weights reflect both relationship frequency (3–9 occurrences) and polarity consistency across independent abstracts, with W = log(F+1) × P.

Analysis of the 4,866 extracted relationships—merged into 4,772 unique weighted edges after duplicates removal—revealed twelve distinct relationship types across three polarity categories (Figure 2a). The most frequent relationship type was associated_with (n = 1,588), reflecting the predominance of observational and correlative findings in diet-microbiome literature. Neutral or mechanistic relationship types also included affects (n = 742), causes (n = 361), regulates (n = 153), modifies (n = 74), maintains (n = 57), and assists (n = 13). Beneficial effects were captured by benefits (n = 720) and increases (n = 381), while harmful effects were captured through reduces (n = 402), inhibits (n = 164), and harms (n = 117). This distribution reflects a mixture of observational human studies and intervention-driven mechanistic investigations characteristic of the field.

Polarity analysis showed that 62.6% of relationships were neutral or observational, 23.1% were beneficial, and 14.3% reflected harmful or inhibitory effects (Figure 2b). This pattern aligns with the dominance of associative evidence in human nutrition studies and the smaller proportion of experimental or interventional findings.

Edge-weight analysis revealed substantial variability in evidence strength across all relationship types (Figure 2c). Beneficial and harmful relationships generally exhibited higher average weights, with benefits (0.844), increases (0.833), harms (0.771), reduces (0.765), and inhibits (0.762) ranking highest. Mechanistic relationship types showed moderate average weights, including regulates (0.707) and affects (0.706). Observational or less-specific relationships—such as associated_with (0.700), modifies (0.699), causes (0.694), assists (0.693), and maintains (0.693)—displayed lower average weights, consistent with more heterogeneous or context-dependent evidence.

At the individual-edge level, the highest-weight relationships included affects_1 (2.30), associated_with_1 (1.95), regulates (1.95), and benefits_1 (1.93) (Figure 2d). Additional high-weight edges comprised benefits_2 (1.93), associated_with_2 (1.79), harms (1.77), benefits_3 (1.66), affects_2 (1.61), and affects_3 (1.39). Indexed labels (e.g., affects_1, associated_with_1) denote distinct source-target entity pairs under the same relationship type; full mappings are provided in Supplementary Table S1. These high-weight edges reflect repeated, polarity-consistent findings across multiple independent abstracts, with frequency counts ranging from 3 to 9 occurrences.

### Network Topology and Modular Structure

The DMH-KG exhibits hallmark structural features of biological networks. Despite low global density—typical of large heterogeneous graphs—the network displays strong local clustering, with a mean clustering coefficient of 0.61. This pattern indicates the presence of densely interconnected neighborhoods within the network.

**Figure 3.**
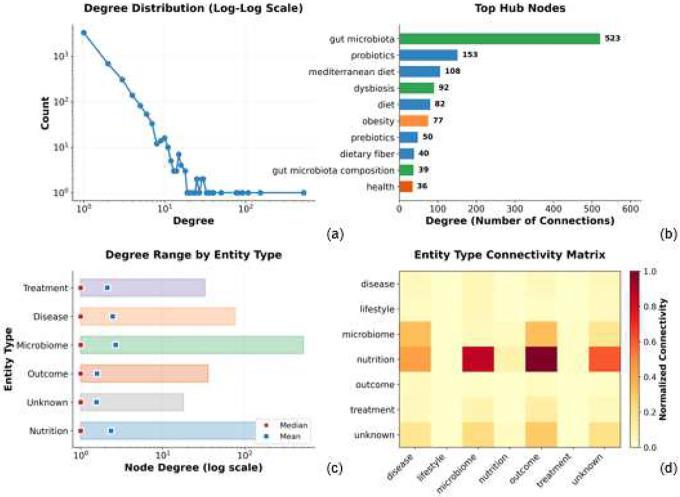
Network topology and structural characteristics of the DMH-KG. The DMH-KG exhibits classic biological network organization. (a) The degree distribution follows a power-law on a log–log scale, with 4,710 nodes spanning degrees 1–523, indicating a scale-free structure dominated by many low-degree entities and a few highly connected hubs. (b) The top 10 hubs—led by *gut microbiota* (degree 523), *probiotics* (153), and *Mediterranean diet* (108)—highlight nodes that integrate evidence across diverse mechanistic contexts, with bar colors denoting entity types. (c) Degree ranges by entity type reveal strongly right-skewed distributions: median connectivity is low (around 1) across categories, but several microbiome and nutrition entities act as major hubs, reflected by wide degree ranges and higher means than medians. (d) The normalized entity-type connectivity matrix shows strongest interactions among nutrition–outcome, microbiome–nutrition, and nutrition–disease pairs, underscoring nutrition’s central mediating role within the diet–microbiome–health axis, while lighter cells represent indirect or sparsely reported connections.

Connectivity patterns further highlight biologically meaningful organization. Nodes with high weighted degree included Faecalibacterium prausnitzii, Akkermansia muciniphila, TMAO, and inflammation, consistent with their recognized roles as keystone taxa or host-response mediators^19^ (Figure 3a–b). These hubs anchor cross-cutting mechanistic pathways spanning metabolism, immune regulation, and diet-driven microbial shifts.

Expert-guided consolidation revealed the Mediterranean diet as a major dietary hub (degree = 108), ranking immediately after gut microbiota (523) and probiotics (153). More than twenty lexical variants—including “Mediterranean diet (MD),” “Mediterranean,” “alternate Mediterranean diet (aMED),” and regional spelling forms—were unified into a single normalized node. This consolidation exposed the diet’s broad mechanistic reach, linking anti-inflammatory pathways, cardiovascular protection, and gut-microbiota modulation.

Degree distributions across entity types (Figure 3c) exhibit strongly right-skewed patterns, with low median connectivity across all categories. Nutrition- and microbiome-related entities exhibited the widest degree ranges and higher means degrees, reflecting a small number of highly connected hubs that integrate evidence across multiple diseases and outcomes. Consistent with this structure, the normalized entity-type connectivity matrix (Figure 3d) revealed dense interactions between nutrition-outcome, microbiome-nutrition, and nutrition-disease pairs, highlighting diet and microbiome concepts as integrative bridges across the network.

### High-Confidence Mechanistic Pathways

**Figure 4.**
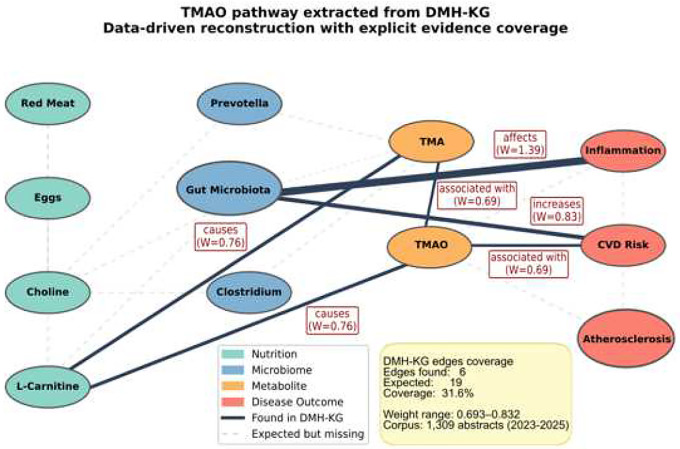
TMAO Pathway Reconstruction as a Bridge Between Quantification and Validation. Data-driven reconstruction of the Choline/Carnitine–Microbiota–TMAO–CVD pathway (Table 1, row 5) validated against Reactome R-HSA-8964043 (Table 2, row 3). Solid arrows indicate relationships found in DMH-KG; dotted lines show expected but missing relationships. Evidence metrics: Pathway quantification (Table 1): 3 documents, 8 relationships; Edge coverage: 6/19 relationships detected (31.6%); Entity coverage: 12/13 pathway markers detected (92.3%, Table 2). Node colors: nutrition (cyan), microbiome (blue), metabolite (orange), disease outcome (red). Edge thickness proportional to evidence weight. Edge coverage measures explicit relationship mentions in literature; entity coverage measures detection of predefined pathway markers from curated databases (KEGG/Reactome).

Systematic traversal of the evidence-weighted DMH-KG identified recurrent, biologically coherent mechanistic pathways linking dietary exposures to host outcomes through microbial and metabolic intermediates (Table 1). These pathways were operationally defined as directional chains spanning Diet → Microbiome → Metabolite → Host Outcome, capturing causal or modulatory relationships consistently reported across the literature. Table 1 summarizes the identified pathways alongside quantitative evidence metrics. One representative pathway—the TMAO formation cascade—is visualized in detail in Figure 4.

Among beneficial mechanisms, the Fiber-Faecalibacterium-ButyrateInflammation axis was supported by 36 publications and 41 relations (Table 1). This pathway reflects the established role of fermentable dietary fiber in promoting butyrate-producing commensal, enhancing intestinal barrier integrity, and reduced inflammatory signaling^20^. A closely related beneficial mechanism, the Probiotic-SCFA-Immunity axis, supported by 34 documents and 43 relations, describes how Lactobacillus and Bifidobacterium supplementation enhances SCFA production and immune regulation^21^.

In contrast, the dominant harmful pathway identified was the High-Fat Diet-LPS-Metabolic Endotoxemia cascade, supported by 8 publications and 11 relations (Table 1). This pathway links high-fat dietary patterns to microbiome dysbiosis, increased lipopolysaccharide translocation, and downstream insulin resistance, closely mirroring the concept of metabolic endotoxemia^22^.

The Choline/L-carnitine-Gut microbiota-TMAO-Cardiovascular disease pathway provides a representative example of the DMH-KG’s dual validation strategy (Figure 4). Supported by 3 publications and 8 extracted relations, this pathway integrates dietary precursors (choline, L-carnitine), microbial intermediates (e.g., Prevotella, Clostridium), metabolites (TMA, TMAO), and cardiovascular outcomes^23,24^. High entity coverage (92.3%) contrasted with moderate edge coverage (31.6%), reflecting the need to synthesize distributed evidence across multiple studies to reconstruct complete multi-step mechanisms.

These analyses illustrate a dual validation strategy applied to the DMH-KG. High-confidence pathways were first identified through data-driven aggregation of literature-supported relationships (Table 1) and subsequently benchmarked against curated KEGG and Reactome pathway definitions. Figure 4 uses the TMAO pathway as a representative example, demonstrating both internal evidence aggregation and external pathway-level validation. These validated mechanisms extend beyond gut-localized effects and intersect with systemic metabolic and immune processes, whose relevance to aging is further explored in the Discussion.

### Quantitative Validation against Curated Pathways

To quantitatively assess alignment between the DMH-KG and established biological knowledge, we performed a structured validation against five metabolic and signaling pathways from KEGG and Reactome (Table 2). These pathways were selected to represent distinct diet-microbiome-health mechanisms and included Butanoate Metabolism (KEGG map00650), Secondary Bile Acid Biosynthesis (KEGG map00121), Tryptophan Metabolism (KEGG map00380), Amine-derived Hormones (Reactome R-HSA-8964043), and Toll-Like Receptor Cascades (Reactome R-HSA-168898).

For each pathway, a predefined set of marker entities was compiled from the corresponding KEGG or Reactome pathway definitions. Marker sets included dietary components, microbial taxa, metabolites, host signaling molecules, and disease or outcome terms, yielding between 11 and 14 reference entities per pathway. Entity coverage was calculated as the proportion of predefined pathway markers that were present as nodes in the DMH-KG.

The DMH-KG achieved complete entity coverage (100%) for both Butanoate Metabolism (14/14 markers) and Secondary Bile Acid Biosynthesis (13/13 markers). Coverage for the Amine-derived Hormones pathway, representing the TMAO mechanism, was 92.3% (12/13 markers). For Toll-Like Receptor Cascades, 85.7% of pathway markers were recovered (12/14), while Tryptophan Metabolism showed 81.8% coverage (9/11).

Across all five pathways, the unweighted mean entity coverage was 92.0%, with coverage values ranging from 81.8% to 100% (Table 2). These results quantify the extent to which predefined pathway components from curated biological databases are represented within the DMH-KG. Complete marker definitions, external pathway mappings, and validation details are provided in Supplementary Table S3.

## Discussion

This study presents a structured, evidence-based framework for decoding the complex interactions linking diet, the gut microbiome, and human health. Through integrating knowledge from recent literature into a polarity-aware DMH-KG, the framework integrates fragmented findings into a unified semantic representation that preserves directionality, microbial mediation, and host outcomes. In contrast to co-occurrence-based resources, the DMH-KG enables retrieval of multi-step mechanistic chains grounded in explicit evidence rather than isolated associations.

### Transforming Unstructured Biomedical Text into Mechanistic Evidence

A significant contribution of this work lies in the development of a domain-precise and interpretable pipeline that transforms narrative biomedical text into structured mechanistic evidence. Expert-guided consolidation resolved lexical fragmentation, amplifying the true biological signal. For example, consolidating variants such as gut microbiome, intestinal flora, and intestinal microbiota enabled the diet-microbiota relationships to emerge as dominant signals supported by multiple independent studies, rather than remaining dispersed across weak, fragmented edges. This consolidation step was essential for revealing consensus mechanisms distributed across the literature.

In addition, explicit encoding of relationship polarity—beneficial, harmful, or neutral—addresses a key limitation of many existing biomedical knowledge graphs, which often lack the semantic resolution required for clinical or nutritional interpretation. Polarity-aware weighting enables clear differentiation between health-promoting mechanisms (e.g., fiber → butyrate → reduced inflammation) and pathogenic cascades (e.g., high-fat diet → LPS → endotoxemia). This design preserves biological direction and provenance while supporting transparent mechanistic reasoning.

### Mechanistically Grounded Knowledge Graphs as Complements to LLMs

LLMs have substantially expanded access to biomedical knowledge, yet they do not inherently encode causal structure and frequently conflate statistically plausible associations with empirically supported mechanisms^25^. This limitation is particularly consequential in nutrition and microbiome research, where subtle mechanistic distinctions—such as whether a metabolite is microbially derived or host-generated, or whether a dietary fiber promotes butyrogenesis versus exacerbates dysbiosis—carry important clinical and translational implications^13,26,27^.

The DMH-KG help mitigate these limitations by providing an evidence-guided substrate that complements neural generative models. Each relationship is anchored to its originating PubMed identifier and enriched with polarity-weighted evidence, enabling directionally consistent and traceable inference while constraining unsupported generalizations^28–30^. Rather than relying solely on probabilistic text completion, mechanistic queries can be resolved through traversable, lineage-preserving pathways that reflect accumulated empirical support.

In a comparative pilot evaluation (Supplementary Table S2; Box 1), a DMH-KG-augmented system consistently recovered multi-step mechanistic chains—such as dysbiosis → bacterial toxins (B. fragilis toxin, colibactin) → epithelial barrier dysfunction → systemic inflammation → age-related diseases—that a standalone LLM either omitted or reduced to generic statements. These findings underscore the value of neurosymbolic reasoning frameworks that integrate structured mechanistic knowledge with the expressive capabilities of LLMs, supporting both interpretability and mechanistic completeness^18^.

### Network Organization and Evidence Asymmetry

The emergent topology of the DMH-KG exhibits organizational properties characteristic of biological systems, including a right-skewed degree distribution and high clustering coefficient (0.61). Such features are well documented in metabolic, ecological, and signaling networks, where a limited number of highly connected components coordinate information flow across specialized functional subsystems.

Network topology analysis further supports the biological plausibility of the DMH-KG. Highly connected entities such as Faecalibacterium prausnitzii, Akkermansia muciniphila, TMAO, and inflammation occupy integrative positions within the network, consistent with their recognized roles as keystone taxa or host-response mediators^31,32^. These nodes act as convergence points linking dietary exposures, microbial transformations, and downstream host outcomes.

At the same time, the network reveals asymmetries in the current evidence base. Beneficial mechanisms—particularly those involving dietary fiber, probiotics, and SCFA production—are densely represented, reflecting both research emphasis and publication bias. In contrast, potentially harmful or unexplored processes, including xenobiotic metabolism, artificial sweeteners, food additives, and other non-nutritive dietary exposures, are comparatively sparse. These gaps indicate areas where mechanistic understanding remains limited and where targeted experimental and clinical studies may be most informative.

As illustrated in Figure 4, individual abstracts rarely describe the complete multi-step TMAO pathway in isolation; however, the evidence-weighted consolidation enables reconstruction of the full mechanistic chain linking dietary choline/L-carnitine to microbial metabolism and cardiovascular outcomes. This illustrates how the DMH-KG bridges data-driven pathway discovery (Table 1) with expert-curated benchmarking (Table 2). The complementary entity- and edge-coverage metrics highlight that pathway components are well represented, while complete mechanistic chains emerge through cross-study synthesis.

### Implications for Precision Nutrition and Clinical Translation

The mechanistic insights encoded in the DMH-KG have direct implications for precision nutrition, where effective interventions depend not only on identifying beneficial dietary components but on understanding how these components act through specific microbial and host pathways. For instance, the *fiber* → *Faecalibacterium* → *butyrate* → *barrier* integrity chain provides a computable rationale for selecting targeted fermentable substrates, such as inulin or resistant starch, in conditions characterized by impaired gut barrier function, including inflammatory bowel disease and metabolic endotoxemia. Similarly, the *polyphenols* → *Akkermansia* pathway highlights diet-microbe interactions that are increasingly recognized as therapeutic targets in obesity and metabolic disorder^33,34^.

Beyond nutritional insight, the DMH-KG addresses key translational challenges associated with the use of generative AI in clinical and dietary counselling. By encoding polarity-aware, evidence-weighted mechanistic relationships with explicit provenance, the graph provides auditability and interpretability that are essential for safe clinical deployment. When incorporated into hybrid retrieval-augmented or neurosymbolic architectures, the DMH-KG can guide LLM outputs toward verifiable biological mechanisms, reducing hallucination risk and supporting personalized dietary recommendations informed by microbial function and host physiology^28,35^.

### Limitations and Future Directions

This study has limitations inherent to literature-based extraction. First, the DMH-KG reflects publication bias, with beneficial pathways more frequently reported than harmful or null findings^36,37^. Second, the current graph is static and does not capture dose-response effects, time-dependent microbial adaptation, or inter-individual variability. Third, entity and relationship annotation was performed at the abstract level, which may omit mechanistic details available only in full-text articles. While entity coverage across validated pathways is high, moderate edge coverage highlights that mechanistic links are often distributed across multiple publications rather than fully documented within single studies.

Beyond degree-based topology and pathway reconstruction, future extensions of the DMH-KG could incorporate formal community detection methods to identify higher-order functional modules. Algorithms such as the Louvain method, which are widely used to reveal modular structure in biological networks^38,39^, may help delineate coordinated diet-microbiome-health subsystems in larger or dynamically updated versions of the graph.

Future iterations will focus on three complementary expansions:

#### Multi-Omics Integration.

Integrating patient-specific metagenomic and metabolomic data layers onto the graph nodes facilitates the creation of digital twins of the gut ecosystem. This direction aligns with the integrative personal omics profile (iPOP) framework, which has demonstrated the efficacy of longitudinal molecular profiling in predicting disease onset and progression^40–42^.

#### Temporal Modelling.

Integrating time-series data and causal representation learning would enable explicit modelling of how dietary interventions reshape the microbiome across different temporal scales. Unlike static snapshots, temporal graph models can distinguish short-term microbial responses (e.g., acute fiber-induced shifts) from the sustained ecological restructuring required to resolve chronic dysbiosis^43,44^.

#### Automated Scalability.

The curated corpus provides a gold standard resource for fine-tuning domain-specific LLMs. This creates a virtuous cycle where the DMH-KG supports automated Named Entity Recognition (NER) and Relation Extraction (RE) with improved precision, enabling large-scale ingestion of full-text literature^17,28,45^. Such automation would allow knowledge graphs to evolve into a living resource that updates continuously as new evidence emerges.

Overall, these features position the DMH-KG as a scalable, evidence-guided platform for mechanistic inference across diet-microbiome-health interactions. By preserving polarity, provenance, and biological direction, the resource supports both discovery-oriented research and AI-driven nutritional reasoning. Continued integration of multi-omics data and longitudinal dietary evidence is expected to further enhance its value for mechanistic hypothesis generation and precision nutrition. Future extensions will explore the incorporation of full-text literature, clinical trial databases, and patient-specific nutritional records to enrich the graph’s granularity and translational relevance—enabling more precise, evidence-grounded reasoning in real-world dietary interventions.

Notably, several high-confidence mechanisms captured by the DMH-KG—such as SCFA-mediated anti-inflammatory signaling and LPS-associated endotoxemia—are directly implicated in immune decline and metabolic dysfunction during aging. These pathways connect to processes including inflammaging, gut barrier disruption, and immunosenescence. The DMH-KG also captures gut-muscle axis relationships, including the association between gut microbiota alterations and sarcopenia, as well as beneficial effects of fiber and SCFAs on muscle metabolism. Structured mechanistic synthesis of literature may therefore help guide nutritional strategies that target immune resilience and metabolic stability during aging. As the gut-immune-brain axis gains prominence in geroscience, the DMH-KG offers a semantically grounded framework for tracing dietary influences on resilience, functional decline, and longevity.

## Methods

### Literature Corpus Curation and Annotation Protocol

We curated a corpus of 1,309 PubMed abstracts using targeted Boolean search strategies designed to capture high-relevance literature at the intersection of diet, the gut microbiome, and human health. The PubMed search strategy employed the following Boolean query: ((“diet” OR “dietary” OR “nutrition” OR “food”) AND (“gut microbiome” OR “gut microbiota” OR “intestinal microbiota”) AND (“health” OR “disease” OR “metabolism”)). Search was conducted using free-text keywords, with PubMed’s automatic MeSH term mapping applied where available. Inclusion criteria required abstracts to report original research or systematic reviews and to be published in English between January 2023 and March 2025. Exclusion criteria included conference abstracts and meeting proceedings without full peer review. Each abstract underwent rigorous manual annotation following a domain-specific guideline developed for the Nutrition-Microbiome-Disease domain, incorporating best practices from biomedical annotation frameworks^46,47^. The schema defined eleven entity categories and twelve directional relationship types, with each relationship additionally assigned a polarity label (beneficial, harmful, or neutral).

A trained annotator (E.B.) completed primary annotation using MedTator^46^, and a senior expert panel (J.L. and C.T.) performed calibration checks before full-scale annotation. This two-step process minimized ambiguity in entity boundaries, directionality, and polarity assignment.

### Knowledge Graph Construction and Integration

Annotated entities and relationships were processed using a systematic consolidation pipeline. During integration, expert-guided synonym normalization was applied to unify synonymous or lexically variant expressions, following established best practices for biomedical knowledge graph construction from heterogeneous literature sources^48,49^; for example, SCFA and short-chain fatty acids were merged, as were conceptually equivalent terms such as intestinal flora, gut microbiota, and gut microbiome, ensuring accurate aggregation of evidence across studies.

Entities not involved in any relationship edges (for example, isolated mentions without connections to other concepts) were excluded to ensure that the constructed graph included only relationally connected, interpretable biological concepts.

To prioritize robust biological associations, we applied a composite edge-weighting function incorporating both evidence frequency and relationship polarity. Each edge weight was calculated as Formula (1):

(1) $$W=log\;\left(F+1\right)\times P$$

where *F* denotes the frequency of the relationship across unique abstracts (log-normalised to stabilise the influence of high-frequency associations), and *P* is polarity multiplier (1.2 for beneficial, 1.1 for harmful, 1.0 for neutral).

### Network topology and Hub Identification

Network topology analysis was performed to characterize the structural organization of the DMH-KG and identify biologically meaningful connectivity patterns. Degree, clustering coefficient, and network density were calculated using standard methods in Python 3.10. Degree was defined as the number of incoming and outgoing edges per entity, with high-degree nodes interpreted as hubs. Network density was computed as the ratio of observed edges to all possible edges, reflecting global graph sparsity.

To support interpretability, edge weights—defined during graph construction—were used throughout all analyses to reflect the strength and polarity consistency of literature-derived associations. These weights incorporate both the frequency of a given relationship and its directional polarity (beneficial, harmful, or neutral), emphasizing biologically robust interactions.

Entities were ranked by weighted degree to identify major hubs within the network. These high-connectivity nodes often represent integrative biological components, such as microbial mediators or diet-linked metabolic regulators. Degree distributions and clustering patterns were further examined to assess the presence of functionally coherent modules. Densely interconnected neighborhoods, such as those surrounding SCFAs or inflammatory mediators, suggest modular substructures consistent with known biological processes.

All visualizations were generated using Matplotlib (v3.8.4), and data processing was conducted using Pandas (v2.3.3). The consolidated graph DMH-KG served as the basis for all network-level analyses and visual summaries presented in Figure 3.

### Validation Strategy

Validation employed a dual framework to assess the biological coherence of the DMH-KG.

#### Internal Validation.

High-confidence mechanistic pathways were identified through expert-guided keyword-based analysis. Pathway definitions were established based on established biological mechanisms documented in systematic reviews and meta-analyses, spanning diverse diet-microbiome-health mechanisms including beneficial fermentation pathways, immune modulation mechanisms, and harmful endotoxemia cascades. For each predefined pathway, comprehensive keyword lists were manually curated to capture all pathway components including dietary inputs, microbial mediators, metabolites, and health outcomes. Relationships were identified by searching for these keywords across entity mentions and relationship types in the DMH-KG. Quantitative evidence metrics—including the number of unique supporting documents and extracted relationships—were computed by counting unique PubMed abstracts (DocIDs) and relationship instances matching pathway criteria (Table 1). These metrics provide transparency regarding the strength of literature support underlying each identified mechanism.

#### External Validation.

Pathway coverage was assessed by benchmarking the DMH-KG against five canonical KEGG and Reactome pathways (Table 2): Butanoate Metabolism (KEGG map00650), Secondary Bile Acid Biosynthesis (KEGG map00121), Tryptophan Metabolism (KEGG map00380), Amine-derived Hormones (Reactome R-HSA-8964043), and Toll-Like Receptor Cascades (Reactome R-HSA-168898). For each external pathway, we defined a structured set of marker entities representing key dietary components, microbial taxa, metabolites, and health outcomes.

Entity coverage (*C*_*entity*_) was quantified as the percentage of predefined pathway marker entities explicitly represented in the DMH-KG:

(2) $$C_{\text{entity}}=\left(M_{\text{detected}}/M_{\text{total}}\right)\times 100\%$$

where *M*_*detected*_ is the number of pathway markers found in the DMH-KG and *M*_*total*_ is the total number of predefined markers for that pathway. This metric reflects the comprehensiveness of component representation.

For selected pathways, we additionally computed edge coverage (*C*_*edge*_), which measures the proportion of mechanistic relationships documented:

(3) $$C_{\text{edge}}=\left(R_{\text{found}}/R_{\text{expected}}\right)\times 100\%$$

where *R*_*found*_ is the number of relationships identified in the DMH-KG and *R*_*expected*_ is the total number of expected pathway connections based on curated pathway databases. Edge coverage is complementary to entity coverage: high entity coverage indicates comprehensive component representation, while edge coverage reflects mechanistic link documentation (Figure 4).

This dual-metric approach provides external validation: if the manually annotated DMH-KG reflects established biology, it should recover the majority of predefined pathway components and connections. Complete marker lists and validation mappings are provided in Supplementary Table S3.

## Supplementary Files

This is a list of supplementary files associated with this preprint. Click to download.

- Supplementary.docx

## Figures and Tables

**Table 1.** High-confidence Diet-Microbiome-Health mechanistic pathways identified from the consolidated DMH-KG, with quantitative evidence metrics.

Pathway Theme	Mechanistic Chain	Polarity	Evidence*	Interpretive Summary
Fiber-SCFA-Inflammation	Dietary fiber → *Faecalibacterium prausnitzii* ↑ → Butyrate ↑ → Intestinal barrier ↑ → Inflammation ↓	Beneficial	36 docs 41 rels	Fiber-driven microbial fermentation increases butyrate, strengthening barrier function and reducing inflammatory markers.
Probiotic-SCFA-Immunity	Probiotic intake → *Lactobacillus/Bifidobacterium* ↑ → SCFA ↑ → Immune regulation ↑	Beneficial	34 docs 43 rels	Probiotics enhance SCFA production and support regulatory immune pathways.
High-Fat-LPS-Metabolic Dysfunction	High-fat diet → Dysbiosis → LPS ↑ → Endotoxemia → Insulin resistance ↑	Harmful	8 docs 11 rels	High-fat diets disrupt the microbiome, elevating LPS and impairing metabolic function.
Choline/Carnitine-TMAO-CVD	Choline/L-carnitine → Gut microbiota → TMA → TMAO ↑ → Atherosclerosis ↑ → CVD risk ↑	Harmful	3 docs 8 rels	Microbial conversion of choline and carnitine to TMAO promotes cardiovascular risk.

* Evidence denotes the number of unique supporting documents (docs) and extracted relations (rels) from DMH-KG.

Abbreviations: SCFA, short-chain fatty acids; LPS, lipopolysaccharide.

**Table 2.** Quantitative validation of DMH-KG-derived pathways against five canonical pathways from KEGG and Reactome.

KG Derived Pathway	Key Entities & Relations (Extracted)	External Mapping	Coverage*
Fiber Fermentation	**N:** Fiber/Starch → **M:** *Faecalibacterium* → **O:** Butyrate	KEGG map00650 (Butanoate metabolism)	**100%** (14/14)
Bile Acid Transformation	**N:** Fat/Cholesterol→ **M:** *Clostridium*→ **O:** Secondary BAs	KEGG map00121 (Secondary Bile Acid Biosynthesis)	**100%** (13/13)
TMAO signalling	**N:** Red meat/Choline → **M:** *Prevotella* → **O:** TMAO	Reactome R-HSA-8964043 (Amine-derived hormones)	**92.3%** (12/13)
Inflammation/Immunity	**M:** LPS → **G:** *TLR4* → **O:** Inflammation/Cytokines	Reactome R-HSA-168898 (Toll-Like Receptor Cascades)	**85.7%** (12/14)
Tryptophan Metabolism	**N:** Tryptophan→ **M:** Microbiota → **O:** Serotonin	KEGG map00380 (Tryptophan metabolism)	**81.8%** (9/11)

* Coverage represents the percentage of predefined pathway marker entities detected in the DMH-KG. For each external KEGG or Reactome pathway, we identified key components (dietary factors, microbial taxa, metabolites, disease markers) and quantified how many appear as nodes in the DMH-KG. Entity coverage differs from edge coverage (see Figure 4, TMAO pathway): entity coverage measures component detection, while edge coverage measures relationship mentions. Complete marker lists and validation mappings are provided in Supplementary Table S3.

## Data Availability

The data and scripts supporting the findings of this study are available from the corresponding author upon reasonable request.

## Box 1. Comparative analysis of standard LLM vs. DMH-KG-augmented system for mechanistic reasoning.

To validate the translational utility of the DMH-KG for aging research, we compared a standard LLM (GPT-4o) against the DMH-KG-augmented retrieval system using a representative query.

**Query:** *“How does gut microbiota dysbiosis contribute to inflammaging and age-related diseases?”*

**Table:**

Standard GPT-4o (Baseline)	DMH-KG-Augmented System
**Response Summary:** “Gut microbiota dysbiosis contributes to inflammaging through: (1) increased gut permeability allowing LPS to enter bloodstream; (2) altered immune function promoting chronic inflammation; (3) reduced SCFA production impairing gut barrier; (4) imbalance favoring pro-inflammatory bacteria; (5) metabolic disruption leading to insulin resistance; (6) neuroinflammation via gut-brain axis.” Key mechanisms cited: leaky gut, LPS translocation, reduced SCFAs, immune dysregulation.”	**Mechanistic Insight (with evidence weights):** (1) Toxin Production: Dysbiotic microbiota increases Bacteroides fragilis toxin, colibactin, and cytolethal distending toxin (W: 0.83), promoting epithelial barrier dysfunction. (2) Barrier Dysfunction: Increased intestinal permeability (W: 0.83) enables LPS translocation and systemic inflammation. (3) Metabolite Alterations: Dysbiosis affects gut metabolite profile (W: 1.10), reducing beneficial SCFA production. (4) Disease Associations: Directly linked to aging-related diseases (W: 1.10): IBD, colorectal cancer, obesity, T2D. (5) Interventions: Mediterranean diet (W: 0.83), probiotics/prebiotics (W: 0.83) benefit dysbiosis.
**Critique:** Accurate but generic. Describes broad mechanisms (leaky gut, LPS, SCFAs) without identifying specific bacterial toxins, quantifiable evidence, or actionable microbial targets for intervention.	**Critique:** Actionable and traceable. Identifies specific bacterial toxins (B. fragilis toxin, colibactin, cytolethal distending toxin) with evidence weights, enabling targeted biomarker development and microbiome-informed therapeutic strategies for healthy aging.

Note: DMH-KG-augmented responses include evidence weights (W) reflecting literature support strength. Full retrieved graph triples and additional case studies available in Supplementary Table S2.
